# Bacterial resistance trends among intraoperative bone culture of chronic osteomyelitis in an affiliated hospital of South China for twelve years

**DOI:** 10.1186/s12879-019-4460-y

**Published:** 2019-09-18

**Authors:** Xianghong Zhang, Qiong Lu, Tang Liu, Zhihong Li, Weiliang Cai

**Affiliations:** 1 Department of Orthopedics, The Second Xiangya Hospital of Central South University, 139# Middle Renmin Road, Changsha, 410011 Hunan China; 2 Department of Orthopedics, Liuzhou General Hospital, Guangxi University of Science and Technology, Liuzhou, 545000 Guangxi China; 3 Department of Pharmacy, The Second Xiangya Hospital of Central South University, Changsha, 410011 Hunan China

**Keywords:** Chronic osteomyelitis, Bacterial culture positive rate, Intra-operative bone culture, Long-term secular trends, antibiotic resistance

## Abstract

**Background:**

The purpose of this study was to gather temporal trends on bacteria epidemiology and resistance of intraoperative bone culture from chronic ostemyelitis at an affiliated hospital in South China.

**Method:**

Records of patients with chronic osteomyelitis from 2003 to 2014 were retrospectively reviewed. The medical data were extracted using a unified protocol. Antimicrobial susceptibility testing was carried out by means of a unified protocol using the Kirby-Bauer method, results were analyzed according to Clinical and Laboratory Standards Institute definitions.

**Result:**

Four hundred eighteen cases met our inclusion criteria. For pathogen distribution, the top five strains were *Staphylococcus aureus* (27.9%); *Pseudomonas aeruginosa* (12.1%); *Enterobacter cloacae* (9.5%); *Acinetobacter baumanii* (9.0%) and *Escherichia coli* (7.8%). Bacterial culture positive rate was decreased significantly among different year-groups. Mutiple bacterial infection rate was 28.1%. One strain of *Staphylococcus aureus* was resistant to linezolid and vancomycin. Resistance of *Pseudomonas aeruginosa* stains to Cefazolin, Cefuroxime, Cefotaxime, and Cefoxitin were 100% nearly. Resistance of *Acinetobacter baumanii* stains against Cefazolin, Cefuroxime were 100%. Ciprofloxacin resistance among *Escherichia coli* isolates increased from 25 to 44.4%. On the contrary, resistance of *Enterobacter cloacae* stains to Cefotaxime and Ceftazidime were decreased from 83.3 to 36.4%.

**Conclusions:**

From 2003 to 2014, positive rate of intraoperative bone culture of chronic osteomyelitis was decreased; the proportion of *Staphylococcus aureus* was decreased gradually, and our results indicate the importance of bacterial surveilance studies about chronic osteomyelitis.

## Background

Osteomyelitis, as a serious deep bone infection, is caused by microorganisms [1, 2], and persistence of microorganisms, low-grade inflammation are chronic osteomyelitis characteristics [3, 4]. Trueta J demonstrated that hematogenous osteomyelitis was caused by a single agent, while other mechanisms of infection showed poymicrobial infection [5]. Hematogenous osteomyelitis was considered as predominantly pediatric disease with 85% of patients aged below 17 years, while about 47–50% of all osteomyelitis was post-traumatic in adult patients [6]. Staphylococcus species was the most common isolated microorganism in most types of osteomyelitis, approximately affecting 50–70% of cases [7, 8], and the second and third were Enterobacteriaceae and Pseudomonas species [9]. Meanwhile, the treatment of chronic osteomyelitis remains challenge, and multidisciplinary approach including adequate surgery and antibiotics were required [10, 11]. Though microbiologic testing would cause false-negative result, it’s an useful mean to identifying the organism [12], and intra-operative bone culture appears to predict the complete etiologic organisms more reliably [13].

Osteomyelitis’ commonly isolated microorganisms were related to age and susceptibility factors, which including injectable drug users, immunocompromised, urinary infection, orthopedic fixation devices, diabetes mellitus and so forth [14–16]. With the rapid development of antimicrobial resistance and expression of virulence factors, regardless of patient’s immune status, the bacterial distribution, bacterial culture positive rate and antibiotic resistance of osteomyelitis had changed gradually [17]. There was few studies about continuous changes of bacterial culture positive rate, causative organisms and antibiotic resistance for intraoperative bone culture from chronic osteomyelitis in the same hospital over a period of time. Up to now, few study was from mainland China, a developing country. Our present study was aimed to evaluate the changing trends of positive rate, causative organisms and antibiotic resistance for intraoperative bone culture from chronic osteomyelitis over a 12-year periods in a south-central region of China. Our this study can help to see the status of bacterial culture positive rate of intraoperative bone and antimicrobial resistance of causative organisms of chronic osteomyelitis, even can help to take more effective measures for treatment.

## Methods

We retrospectively reviewed the medical records of patients who were admitted to the orthopedics department with chronic osteomyelitis from January 12,003, to December 31,2014. The health facility is an university teaching hospital that located in the south central region of China. Medical record information included the basic information of patients, cause of osteomyelitis, the bone(s) affected, the status of bacterial culture, antimicrobial susceptibility testing, results of laboratory tests and radiography, and even pathological examination. Chronic ostemyelitis was defined clinically as bone infection with clinical signs persisting for more than 10 days or the relapse of a previously treated or untreated osteomyelitis [18], and bone infection was defined as at least two bone cultures with the same organism growth, or one positive bone culture combined with the intraoperative finding of purulence, acute inflammation on histologic examination consistent with infection, or a sinus tract communicating to the bone [19]. Patients were choose in accordance with the unified standards [20]. At first, we picked over cases with chronic osteomyelitis which were diagnosed based on above definition through the medical records; and then chronic osteomyelitis who had taken intraoperative bone culture was sorted out; Lastly, we had shut out cases who had not quit antibiotic therapy for at least 1 week within the period preceding admission for surgery. In order to avoid duplicate counts, only one isolate from the same species was included per patients [21]. Specimens from the depths of sinus tracks were taken for surgery. Marrow pus, curetting, sequestra and bone biopsy were obtained at surgery, and sent through appropriate transport medium for microbiological examination and culture. All samples were inoculated onto a pair of blood agar and one Mac Conkey agar plates. One blood agar plate was inoculated anaerobically for 48 h and the other two plates aerobically for 24 h. Special identification of the isolates was performed by standard biochemical methods, and antimicrobial susceptibility testing was carried out by means of a unified protocol using the Kirby-Bauer method, results were analyzed according to Clinical and Laboratory Standards Institute (CLSI) criteria (as applicable each year) [22].

Statistical analysis was performed with the Statistical package for social sciences (SPSS)21.0 software (SPSS Inc., Chicago, IL, USA). Patient’s demographics were described as the mean and the standard deviation or as the count and percentage as appropriate. Chi-square test or Kruskal-Wallis H test was used to analysis the difference of rates about numeration data. All significance tests were two-sided, and *p* value of less than 0.05 was considered statistically significant for all tests.

## Results

In order to increase the number of different group, we divided 2 years into one group. The mean age was 39.3 ± 16.5 years, and 10.8% of the patients was younger than 18 years, 50.7% was 18–45 years, and 38.5% of the cohort was 45 years or older; and 327 of patients were males (78.2%). The majority of the infections (95.7%) involved only one bone, and the most common anatomical affected sites was tibia (35.9%); followed by femur (27.5%); calcaneus (5.7%) and humerus (2.2%). We have observed an increasing proportion of culture-negative (28.5%), and positive rates were statistically different between all year-groups (χ^2^ = 11.95, *P* = 0.036). Age-specific and Etiology-specific positive rate for intraoperative bone culture were shown in Tables 1 and 2. The year-trend of positive rate was not significantly different in the age-group of younger than 18 years(*p* = 0.062) and group of 45 years or older (*p* = 0.117), but were statistically different in the group of 18–45 years (*p* = 0.003). In the risk-factors of different etiology, positive rate was statistically different in traumatic-group (*p* = 0.00) and hematogenic-group (*p* = 0.01).

**Table 1:** Age-specific positive rate for intraoperative bone culture of chronic osteomyelites (2003–2014)

	Positive-rate(%)		
Age group (yr)	< 18	18–44	≥45
Year group (yr)			
2003–2004	25.0	46.4	41.2
2005–2006	55.6	49.1	61.9
2007–2008	60.0	68.1	66.7
2009–2010	75.0	70.0	62.5
2011–2012	80.0	80.0	78.2
2013–2014	66.7	70.1	67.8
H value	10.530	18.270	8.811
*P* value	0.062	0.006*	0.117

^*^P < 0.05 statistically significant

**Table 2:** Etiology-specific positive rate for intraoperative bone culture of chronic osteomyelites (2003–2014)

Pathogenesis	Positive-rate(%)			
	traumatic	adjacent infection	hematogenic	others
Year group				
2003–2004	53.8	16.7	27.31	35.7
2005–2006	50.9	37.5	57.1	66.7
2007–2008	68.4	55.6	100.0	63.6
2009–2010	69.1	66.7	80.0	61.3
2011–2012	88.7	70.6	90.0	64.3
2013–2014	79.7	50.0	100.0	59.0
H value	28.081	6.900	16.686	4.191
*P* value	0.000^*^	0.228	0.005^*^	0.522

^*^P < 0.05 statistically significant

**Fig. 1:**
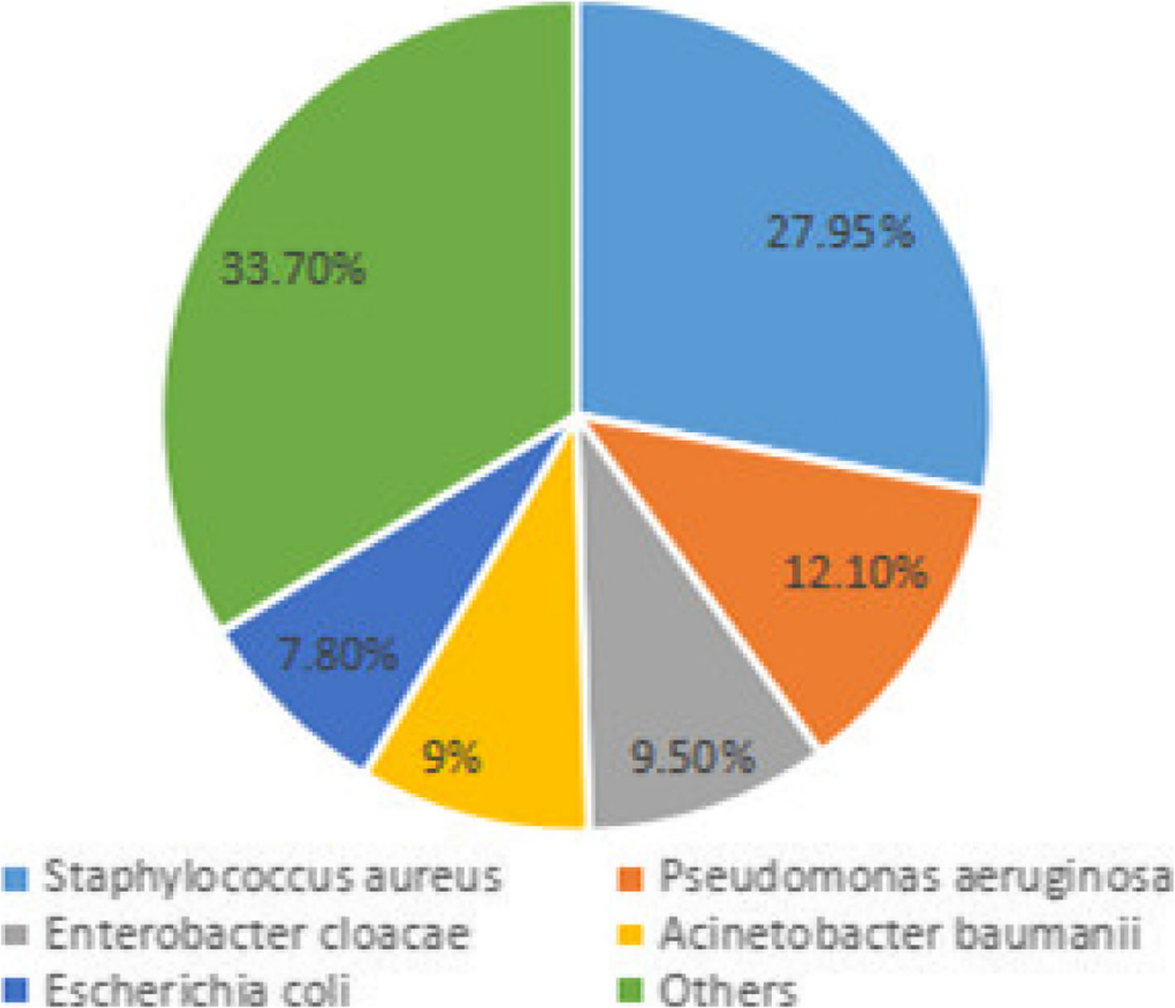
The pathogen distribution of chronic osteomyelitis from 2003 to 2014

The total number of bacterial isolated from 418 cases was 398. The percentage of the top five species was shown in Fig. 1: *Staphylococcus aureus* infections was responsible for 27.9%, followed by *Pseudomonas aeruginosa* (12.1%); *Enterobacter cloacae* (9.5%); Acinetobacter baumanii (9.0%) and *Escherichia coli* (7.8%). Mutiple bacterial infection rate was 28.1%, which included three strains infection rate (5.02%) and double bacterial infection rate (23.08%). For *Staphylococcus aureus*, We found one strain in group of 2009–2010, which was resistant to linezolid; and found one strain in group of 2011–2012 resistant to vancomycin and linezolid. Ciprofloxacin and Erythromycin resistance levels decreased from 41.7 to 30% and from 66.7 to 46.7% (Table 3). For *Pseudomonas aeruginosa*, Cefazolin, Cefuroxime, Cefotaxime, and Cefoxitin resistance levels were 100% nearly (Table 4). For *Enterobacter cloacae*, We found nearly all resistant to Cefazolin, Cefuroxime, Cefoperazone and Cefoxitin; Cefotaxime and Ceftazidime resistance levels decreased from 100 to 54.5% and from 83.3 to 36.4%, respectively (Table 5). For Acinetobacter baumanii, they were stable 100% nearly to Cefazolin and Cefuroxime (Table 6). For *Escherichia coli*, Gentamicin resistance levels decreased from 50 to 22.2%, respectively. However, a marked increase of resistance was seen for Ciprofloxacin from 25 to 44.4%, respectively (Table 7).

**Table 3:** Resistance rates(%) of *Staphylococcus aureus* to antimicrobial agents

Antimicrobial agent	2003–2004 (*n* = 5)	2005–2006 (*n* = 13)	2007–2008 (*n* = 11)	2009–2010 (*n* = 17)	2011–2012 (*n* = 35)	2013–2014 (*n* = 30)
Vancomycin	0	0	0	0	2.9	0
Linezolid	0	0	0	6.3	2.9	0
Teicoplanin	–	0	0	0	0	0
Rifampin	40.0	12.5	0	10.0	25.0	20.0
Levofloxacin	–	–	0	43.8	54.3	33.3
Ciprofloxacin	0	41.7	10.0	26.7	25.7	30.0
Gentamicin	20.0	46.2	0	26.7	34.3	31.0
Clindamycin	–	36.4	10.0	30.8	62.9	44.8
Erythromycin	40.0	66.7	14.3	36.4	65.7	46.7
Penicillin G	33.3	100.0	81.8	87.5	94.3	88.0
Ampicillin	–	100.0	90.0	92.3	96.0	95.8

**Table 4:** Resistance rates(%) of *Pseudomonas aeruginosa* to antimicrobial agents

Antimicrobial agent	2003–2004 (*n* = 1)	2005–2006 (*n* = 8)	2007–2008 (*n* = 3)	2009–2010 (*n* = 7)	2011–2012 (*n* = 14)	2013–2014 (*n* = 15)
Amikacin	0	25.0	0	14.3	14.3	0
Gentamicin	0	25.0	33.3	42.9	28.6	13.3
Piperacillin	0	37.5	66.7	28.6	15.4	14.3
Piperacillin/taazobactam	–	20.0	66.7	28.6	21.4	0
Cefazolin	100.0	100.0	100.0	100.0	100.0	100.0
Cefuroxime	100.0	100.0	–	100.0	100.0	100.0
Cefotaxime	–	100.0	0	75.0	100.0	100.0
Ceftazidime	0	12.5	66.7	14.3	14.3	6.7
Cefepime	–	0	0	28.6	14.3	0
Cefoxitin	100.0	100.0	100.0	–	–	100.0
Imipenem	0	0	33.3	14.3	7.7	6.7
Meropenem	–	0	50.0	66.7	27.3	0
Ciprofloxacin	0	37.5	0	14.3	21.4	13.3

**Table 5:** Resistance rates(%) of *Enterobacter cloacae* to antimicrobial agents

Antimicrobial agent	2003–2004 (*n* = 4)	2005–2006 (*n* = 2)	2007–2008 (*n* = 12)	2009–2010 (*n* = 6)	2011–2012 (*n* = 3)	2013–2014 (*n* = 11)
Amikacin	50.0	0	45.5	33.3	0	9.1
Gentamicin	50.0	100.0	66.7	66.7	66.7	45.5
Piperacillin	50.0	100.0	100.0	83.3	100.0	63.6
Cefazolin	100.0	100.0	100.0	100.0	100.0	100.0
Cefuroxime	75.0	100.0	90.9	66.7	100.0	100.0
Cefotaxime	–	100.0	90.9	83.3	100.0	54.5
Ceftazidime	50.0	0	83.3	83.3	33.3	36.4
Cefepime	100.0	50.0	66.7	66.7	100.0	45.5
Cefoperazone	100.0	100.0	100.0	–	–	100.0
Cefoxitin	100.0	50.0	100.0	–	–	100.0
Imipenem	0	0	0	0	0	9.1
Meropenem	0	0	33.3	–	–	18.2
Ciprofloxacin	0	100.0	54.5	66.7	100.0	9.1

**Table 6:** Resistance rates(%) of *Acinetobacter baumanii* to antimicrobial agents

Antimicrobial agent	2003–2004 (*n* = 0)	2005–2006 (*n* = 3)	2007–2008 (*n* = 6)	2009–2010 (*n* = 8)	2011–2012 (*n* = 17)	2013–2014 (*n* = 2)
Amikacin	–	66.7	66.7	50.0	64.7	100.0
Gentamicin	–	100.0	100.0	75.0	82.4	100.0
Piperacillin	–	100.0	66.7	75.0	88.2	100.0
Piperacillin/taazobactam	–	100.0	100.0	75.0	88.2	100.0
Cefazolin	–	100.0	100.0	100.0	100.0	100.0
Cefuroxime	–	100.0	100.0	100.0	100.0	–
Cefotaxime	–	100.0	80.0	75.0	82.4	100.0
Ceftazidime	–	100.0	60.0	50.0	76.5	50.0
Cefepime	–	100.0	100.0	50.0	70.6	50.0
Imipenem	–	33.3	16.7	50.0	64.7	100.0
Meropenem	–	100.0	0	80.0	75.0	100.0
Ciprofloxacin	–	100.0	83.3	62.5	70.6	100.0

**Table 7:** Resistance rates(%) of *Escherichia coli* to antimicrobial agents

Antimicrobial agent	2003–2004 (*n* = 0)	2005–2006 (*n* = 4)	2007–2008 (*n* = 3)	2009–2010 (*n* = 7)	2011–2012 (*n* = 8)	2013–2014 (*n* = 9)
Amikacin	–	25.0	0	14.3	25.0	0
Gentamicin	–	50.0	100.0	57.1	50.0	22.2
Piperacillin	–	100.0	100.0	100.0	87.5	88.9
Cefazolin	–	50.0	100.0	100.0	75.0	88.9
Cefuroxime	–	75.0	100.0	100.0	75.0	100.0
cefotaxime	–	25.0	100.0	100.0	75.0	77.8
Ceftazidime	–	0	100.0	100.0	75.0	33.3
Cefepime	–	0	100.0	100.0	75.0	66.7
Ciprofloxacin	–	25.0	66.7	100.0	50.0	44.4

## Discussion

A culture directed antibiotic therapy is proper treatment of chronic osteomyelitis [23]. The culture of material should obtained by superficial swabbing of the wound, depths of sinus track, intraoperative and even specimens by other methods. Culture of superficial material swabbing of the would was considered adequate to identify pathogens causing osteomyelitis before 1978 [24], but recent literature have suggested that bone specimen cultures were more reliable compared to sinus track culture on the complete etiologic organisms [20]. Therefore, we taken patients who had made intraoperative bone cultures into our these studies for the aim of gathering temporal trends. Although all kinds of organisms, including bacteria, viruses, parasites, fungi, and tuberculosis may cause osteomyelitis, bone infection was mainly caused by pyogenic bacteria and mycobacteria. Tong SY had drawn a conclusion that *Staphylococcus aureus* was responsible for 80 to 90% of the cases of pyogenic osteomylitis, while *Staphylococcus epidermidis* was the most abundant skin flora [25]. We have discovered *Staphylococcus aureus* infection was responsible for 27.9% of the total number of cases, followed by *Pseudomonas aeruginosa* (12.1%) and *Enterobacter cloacae* (9.5%). Though *Staphylococcus aureus* was still the most common pathogenic bacteria, the proportion was decreased year by year due to long-time, unreasonable and even abuse using agents and increase of high-energy open fractures [26, 27].

Because chronic osteomyelitis require antibiotics therapy for months to years, therefore, chronic osteomyelitis entails a major financial burden and substantially affects the quality of life. This situation made the accurate identification of pathogen as an absolute cornerstone of antimicrobial therapy. We suggested that the treatment of chronic osteomyelitis according to the microbiological analysis from surgery or bone biopsy. Consistent with temporal trends in the distribution of chronic osteomylitis, we observed a decline in the proportion of patients with gram-positive bacteria infections and an increase in the proportion of cases with culture-negative over time. Some recent studies had described an increase in culture-negative cases because of early antibiotic administration and even rampant use of antibiotics [28]. Failure to incubate anaerobic cultures for sufficient time might also have contributed to the culture-negative rate [29]. Culture-negative specimens may because of tuberculosis and fungal infection which should require further investigation using specialized techniques. Biofilms [30] and failure to recognize small colony variants (SCVs) may cause false-negative culture results [31]. Such bacteria are in a stationary phase of growth because oxygen and glucose are limited in biofilms [32]. Small colony variants (SCVs) were first depicted exceed 100 years, and it was 20 years ago to described the relationship between chronic staphylococcal infection and the presence of SCVs [33]. SCVs figure a very heterogeneous bacterial population found in different staphylococcal species. In fact, SCVs are difficult to recover, to identify and to store. Clinical studies had found that SCVs exhibit so-called phenotypic (or functional) resistance beyond the classical resistance mechanisms by their intracellular lifestyle, and SCVs can often be retrieved from therapy-refractory courses when it was infected [33]. Therefore, we recommended that tissue for culture of aerobic organism, anaerobic organism, tubeculosis and fungal must be obtained during intraoperative in order to identify all the etiologic organisms. Treatment failures in chronic osteomyelitis will then be reduced to the minimum.

The published literature had shown the tibia was the most commonly affected site [28], and ours finding have corroborated that tibia (36.1%) was the common site. Osteomyelitis encompasses a broad spectrum of disease mechanisms, and the three generally accepted categories was hematogenic, contiguous to an adjacent infection focus, and direct bacterial inoculation from a traumatic. Our study discovered that direct bacterial inoculation from traumatic was responsible for 54.3% of all cases, followed by adjacent infection (8.6%); hematogenic (8.1%) and others of unexplained factor (28.9%). Along with the advances were made in the management of chronic osteomyelitis, the epidemiology of the condition appears to evolve over time. Chen AT has made a conclusion that the incidence of bone infection may continue to rise because of multiple factors including improved diagnosis, increasing patient risk factors, and increased needs for arthroplasties [34]. Mader JT concluded that the increased survival following traumatic injury has been accompanied by an increased occurrence of post-traumatic osteomyelitis [35]. The patients with post-traumatic osteomyelitis require repeated surgery and long-time agents using, therefore, it may lead to bacterial resistant and lower bacterial culture positive rate.

In our study, we described the trends in the change of positive rate for intraoperative bone culture over time and demonstrated that the positive rate changed substantially over 12-year from 2003 to 2014. We discovered the bacterial culture positive rate for introperative bone was changed by age-factor over time. Age is an important factor which could determine the etiology of chronic osteomyelitis. In children group, the most common etiology was hematogenous infections. Due to elder cases had experienced a higher frequency of disorder that may lead to infection, such as diabetes mellitus, orthopaedic surgeries and vascular or neurologic insufficiency disease, elder patients were susceptible to chronic osteomyelitis. Host condition has been emphasized because it was the importance factor for chronic osteomyelitis treatment modality. Parkkinen M considered host condition-related risk factors for bone infection included diabetes, arteriosclerosis, alcoholism, obesity, smoking, and aging [36]. Therefore, we should make effort on effective prevention and treatment of older’s chronic osteomyelitis since older people are susceptible to be infected and prognosis is poor once chronic osteomyelitis developed.

We made a conclusion that multiple bacterial infection rate was 28.1%, which included three strains infection rate (5.02%) and double bacterial infection rate (23.08%). We found one stain of *Staphylococcus aureus* was resistant to linezolid and vancomycin; and we also found stains of *Pseudomonas aeruginosa*, *Enterobacter cloacae* and Acinetobacter baumanii were resistant to carbapenems. Carbapenems are the most potent and reliable β-lactam antibiotics for the treatment of serious infection caused by multidrug-resistant Gram-negative bacterial [37]. Infection of multidrug-resistant bacterial present a serious clinical challenge for physicians in healthcare setting. Treatment options for these infections are limited, and the use of inappropriate empirical antibiotic therapy of delayed appropriate antibiotic therapy can lead to worse outcomes. We also found large fluctuations over time in our study, and taking antibiotic resistance surveillance studies over longer time periods is important.

We realized that our retrospectively investigation may have been influenced by a number of methodological shortcomings. In this regard, retrospective error is inevitable. We believe that it is important to keep in mind that our study focused on chronic osteomyelitis with intraoperative bone culture and quit antibiotic therapy for at least 1 week. For this reason, the positive rate of intraoperative bone culture may have been higher in these patients in our study in comparison with the patients described in other investigation. Furthermore, the number of cases was distributed uneven each year, although we do not feel that this particular limitation greatly affected the trend of the change of positive rate. Finally, we only made study in a single center in China. Future cohort studies in multi-center study should be taken into research.

## Conclusions

Based on the results of this investigation, the proportion of *Staphylococcus aureus* is decreased gradually, and our results indicate the importance of bacterial surveilance studies about chronic osteomyelitis. Further research is warranted to replicate these findings in more center and to gather temporal trends on bacterial epidemiology and resistance of chronic osteomyelitis.

## Data Availability

The datasets used and/or analysed during the current study are available from the corresponding author on reasonable request.

## Abbreviations

CLSI: Clinical and Laboratory Standards Institute
SCVs: Small colony variants
SPSS: Statistical package for social sciences
